# SARS‐CoV‐2 infection and paediatric endocrine disorders: Risks and management considerations

**DOI:** 10.1002/edm2.262

**Published:** 2021-06-03

**Authors:** Ryan Miller, Ambika P. Ashraf, Evgenia Gourgari, Anshu Gupta, Manmohan K. Kamboj, Brenda Kohn, Amit Lahoti, Daniel Mak, Shilpa Mehta, Deborah Mitchell, Neha Patel, Vandana Raman, Danielle G. Reynolds, Christine Yu, Sowmya Krishnan

**Affiliations:** ^1^ Department of Pediatrics University of Maryland School of Medicine Baltimore MD USA; ^2^ Department of Pediatrics University of Alabama at Birmingham Birmingham AL USA; ^3^ Department of Pediatrics Georgetown University Washington D.C. USA; ^4^ Department of Pediatrics Children's Hospital of Richmond at Virginia Commonwealth University Richmond VA USA; ^5^ Department of Pediatrics Nationwide Children's Hospital The Ohio State University Columbus OH USA; ^6^ Department of Pediatrics NYU Langone Medical Center New York NY USA; ^7^ Department of Pediatrics University of Tennessee Health Sciences Center Le Bonheur Children's Hospital Memphis TN USA; ^8^ Department of Pediatrics New York Medical College New York NY USA; ^9^ Pediatric Endocrine Unit Massachusetts General Hospital for Children Boston MA USA; ^10^ Department of Pediatrics Milton S. Hershey Medical Center Hershey PA USA; ^11^ Department of Pediatrics University of Utah Salt Lake City UT USA; ^12^ Diabetes and Endocrinology Center University of South Florida Tampa FL USA; ^13^ Department of Pediatrics and Department of Medicine University of Chicago Chicago IL USA; ^14^ University of Oklahoma Health Sciences Center Oklahoma City OK USA

**Keywords:** COVID‐19, paediatric endocrine disorders, SARS‐CoV‐2

## Abstract

**Background:**

Coronavirus‐19 (COVID‐19) is a disease caused by the SARS‐CoV‐2 virus, the seventh coronavirus identified as causing disease in humans. The SARS‐CoV‐2 virus has multiple potential pathophysiologic interconnections with endocrine systems, potentially causing disturbances in glucose metabolism, hypothalamic and pituitary function, adrenal function and mineral metabolism. A growing body of data is revealing both the effects of underlying endocrine disorders on COVID‐19 disease outcome and the effects of the SARS‐CoV‐2 virus on endocrine systems. However, comprehensive assessment of the relationship to endocrine disorders in children has been lacking.

**Content:**

In this review, we present the effects of SARS‐CoV‐2 infection on endocrine systems and review the current literature on complications of COVID‐19 disease in underlying paediatric endocrine disorders. We provide recommendations on management of endocrinopathies related to SARS‐CoV‐2 infection in this population.

**Summary and outlook:**

With the surge in COVID‐19 cases worldwide, it is important for paediatric endocrinologists to be aware of the interaction of SARS‐CoV‐2 with the endocrine system and management considerations for patients with underlying disorders who develop COVID‐19 disease. While children and adults share some risk factors that influence risk of complications in SARS‐CoV‐2 infection, it is becoming clear that responses in the paediatric population are distinct and outcomes from adult studies cannot be extrapolated. Evidence emerging from paediatric studies provides some guidance but highlights the need for more research in this area.

## Graphical abstract

The SARS‐CoV‐2 virus has multiple potential pathophysiologic interconnections with endocrine systems. In this review, we present the effects of SARS‐CoV‐2 infection on endocrine systems and review the current literature on complications of COVID‐19 disease in underlying paediatric endocrine disorders. We provide recommendations on management of endocrinopathies related to SARS‐CoV‐2 infection in this population.

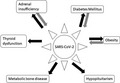

## 1 INTRODUCTION

**FIGURE 1:**
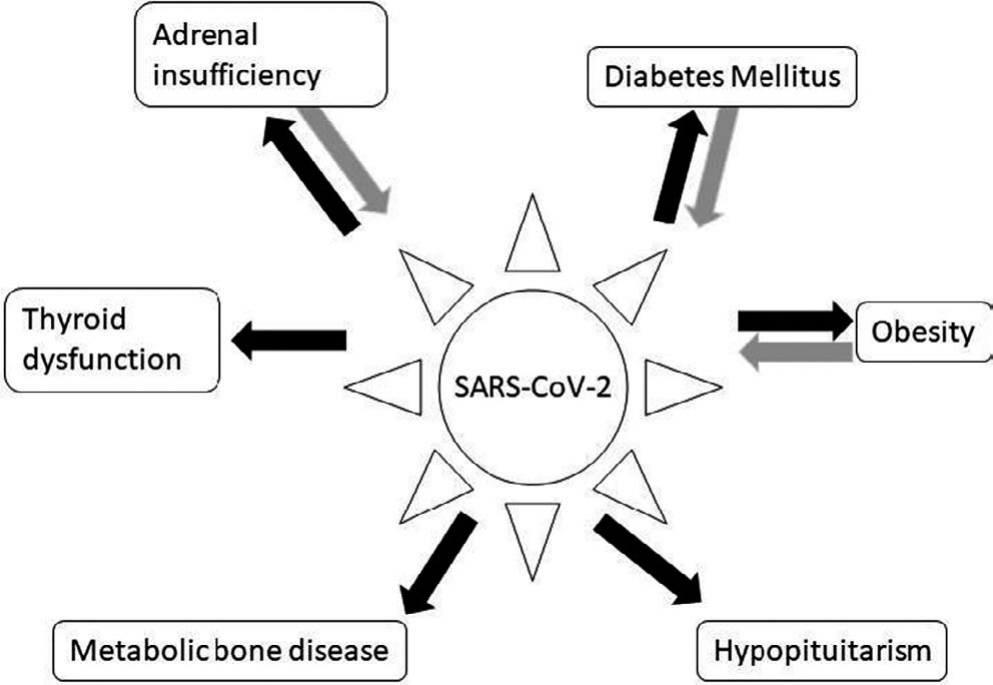
Interactions between SARS‐CoV‐2 infection and endocrine systems

Coronavirus disease 19 (COVID‐19) is caused by the SARS‐CoV‐2 virus, the seventh coronavirus identified as causing disease in humans. Angiotensin‐converting enzyme 2 (ACE2) is considered to be the primary receptor mediating SARS‐CoV‐2 infection.^1^ Binding of SARS‐CoV‐2 to ACE2 triggers a cascade leading to activation of the NF‐kB pathway, increasing proinflammatory cytokines and chemokines to very high levels, leading to the development of acute respiratory distress syndrome (ARDS) seen in severe COVID‐19 disease.^2^, ^3^ Lethality of ARDS and non‐pulmonary complications in COVID‐19 is thought to be due to cytokine storm in which immune and nonimmune cells release large amounts of proinflammatory cytokines that cause damage within and beyond the respiratory system.^4^ A small but growing body of data indicates there may be effects of underlying endocrine disorders on COVID‐19 disease outcome and effects of the SARS‐CoV‐2 virus on endocrine systems (Figure 1).

Children and adolescents were initially thought to experience similar but less severe symptoms and complications of SARS‐CoV‐2 infection.^5^ It is now apparent that children experience unique manifestations of SARS‐CoV‐2 infection, including multisystem inflammatory syndrome (MIS‐C) and distinct endocrine responses.^6^ In this paper, we present what is currently known regarding the effects of SARS‐CoV‐2 infection on endocrine systems, review the current literature on complications of the COVID‐19 pandemic in endocrine disorders and provide recommendations on management of endocrine disorders in children and adolescents with COVID‐19 disease. Most of the clinical literature on SARS‐CoV‐2 and endocrine disorders is in adults; we have attempted to include all paediatric data available in Pubmed as of 31 January 2021. We have included adult data when relevant and have identified when studies refer to adult vs. paediatric study populations. The limited amount of data in children indicates significant differences in response to SARS‐CoV‐2 infection in the paediatric population.^7^ Thus, it is essential for the paediatric endocrine community to understand the manifestations of this disease and the limits of our current knowledge in order to provide optimal care of children in the COVID‐19 era.

## 2 SARS‐COV‐2 INFECTION AND CONSEQUENT ENDOCRINE DYSFUNCTION

The SARS‐CoV‐2 virus has multiple pathophysiologic interconnections with endocrine systems with the potential to cause disturbances in pituitary, adrenal and thyroid function, glucose metabolism and mineral metabolism. Existing data are generally favourable in terms of endocrine complications of COVID‐19 in the paediatric population.

Similarities between COVID‐19, SARS and MERS suggest that the virus may gain access to the central nervous system, including the hypothalamus, via the olfactory bulb.^8^ Observational studies in adults have demonstrated disruption of posterior pituitary function and acute onset of syndrome of inappropriate antidiuretic hormone (SIADH) in COVID‐19.^9^, ^10^, ^11^ Hypothalamic/pituitary dysfunction has been described in SARS survivors.^12^ However, the only evidence of pituitary involvement in COVID‐19 is a finding of pituitary stalk involvement by MRI in two adult patients; to date, there are no reports of pituitary hormone deficiencies in either adults or children with COVID‐19.^13^

There are data to suggest risk of both adrenal and thyroid involvement in adults with COVID‐19. Acutely ill adults with COVID‐19 disease were found to have higher cortisol levels than those without COVID‐19 in one study, but with a reverse correlation between degree of cortisol response and survival rate in those who were COVID‐19‐positive.^14^ Adrenal involvement has also been shown by CT (acute adrenal infarction) and post‐mortem studies in adults with severe COVID‐19 and SARS‐CoV‐2 infection.^15^, ^16^ Both thyrotoxicosis (via association with higher IL‐6 levels) and hypothyroidism have been identified in adults with COVID‐19.^17^, ^18^, ^19^ ACE2 is highly expressed in thyroid tissue, and to a lesser extent in adrenal tissue, while children may theoretically be at risk, thyroid and adrenal disease in children with COVID‐19 and multisystem inflammatory syndrome in children (MIS‐C) have not been reported.^20^

SARS‐CoV‐2 infection may have a diabetogenic effect independent of the stress response associated with severe illness, as ACE2 is highly expressed in pancreatic islet cells.^21^

New onset of diabetes mellitus has been described in adults with COVID‐19 but not children.^22^, ^23^ A global registry established to address questions surrounding the onset of diabetes mellitus in relation to COVID‐19 may determine whether SARS‐CoV‐2 infection is a risk factor for new‐onset diabetes in children and adults.^24^

Somewhat paradoxically, low lipid levels are found in the most severely ill patients with COVID‐19. These patients have very low levels of total cholesterol, LDL and HDL, reflecting an overwhelming inflammatory (cytokine) effect. In recovering ICU patients, lipid levels increase in parallel with decreasing levels of inflammatory markers. While long‐term implications of this phenomenon have not been addressed, suppressed lipid levels, in parallel to elevated inflammatory markers, do appear to denote a worse outcome.^25^, ^26^

Currently, there is no evidence that COVID‐19 directly affects the parathyroid glands or alters mineral ion homeostasis. However, analyses indicate that serum calcium levels can be depressed in adults with severe COVID‐19.^27^, ^28^ There are several reports of hypocalcaemia in paediatric patients with MIS‐C; however, there are no systemic reports in children currently and potential mechanisms have not been addressed.^29^, ^30^

## 3 SARS‐COV‐2 INFECTION AND COMPLICATIONS IN CHILDREN WITH PRE‐EXISTING ENDOCRINE DISORDERS

Data regarding the risks of SARS‐CoV‐2 infection in individuals with underlying endocrine disorders have primarily been described in adults. While findings in adults should not be extrapolated to children, the data do highlight risks to the paediatric population. In this section, we review what is known to date about COVID‐19 in patients with underlying endocrine disorders and how this may impact paediatric patients.

Current evidence does not suggest that central hormone deficiencies increase risk of acquiring SARS‐CoV‐2 infection. However, children and adolescents with multiple pituitary hormone deficiencies present unique management challenges due to the complexity of their medical condition. Infants and children with diabetes insipidus who develop respiratory complications of COVID‐19 have a significantly increased risk of serum sodium abnormalities.^31^ In patients with DI, the risk of hypernatraemia increases in acute illness due to factors including reduced fluid intake, increased insensible losses and inability to tolerate oral desmopressin; adipsic patients with DI are at marked risk of severe hypernatraemia, which may be complicated by venous thrombosis.^32^, ^33^, ^34^, ^35^ As with other viral infections, COVID‐19 is likely to increase risk of adrenal crisis and respiratory complications in patients with adrenal insufficiency (including those on corticosteroid replacement therapy); however, this has not specifically been addressed in either adults or children.

Currently, there are no data indicating increased risk of acquiring SARS‐CoV‐2 infection or altered disease course in children and adolescents with underlying thyroid disorders. However, it is important to keep in mind that patients with Graves' disease treated with anti‐thyroid drug (ATD) therapy are at higher risk of agranulocytosis and secondary infections.^36^ This is particularly important as data from one study showed that half of COVID‐19 non‐survivors experienced a secondary infection.^37^ Underlying thyroid disease, including hypothyroidism, does appear to be a risk factor for a more severe disease course in adults with COVID‐19.^38^, ^39^, ^40^

It has been well documented that adults with diabetes mellitus, obesity and hypertension are at higher risk COVID‐19 infection and experience higher rates of complications and death.^40^, ^41^, ^42^, ^43^, ^44^ The T1D Exchange has published data on 64 adults with T1D; 33 were COVID‐19‐positive and 31 had COVID‐19‐like symptoms but were either not tested or were COVID‐19‐negative. 65.5% of individuals were <19 years of age.^45^ The COVID‐19‐positive group was found to have a higher mean HbA1C (8.5% vs. 8%) were more likely to present in DKA (45.5% vs. 13.3%) and required a higher level of care compared with the COVID‐like group. A population‐based study in England showed that people with an HbA1C of 86 mmol/mol (10.0%) or higher compared with people with an HbA1C of 48–53 mmol/mol (6.5–7.0%) had increased COVID‐19‐related mortality (hazard ratio [HR] 2·23 [95% CI 1·50–3·30, *p* < .0001] in T1D).^46^

T1D Exchange data in the paediatric population demonstrated higher A1C, increased risk of hospitalization, non‐Hispanic Black ethnicity and public insurance in children with T1D and COVID‐19 (unpublished data). Children presenting with new‐onset T1D may also be more likely to present in DKA and may have more severe DKA in during the coronavirus pandemic.^47^ However, evidence to date suggests that children with T1D and COVID‐19 do not have worse disease outcomes than those without diabetes.^48^

Children with diabetes have faced unique challenges related to the COVID‐19 pandemic, primarily related to widespread closures of schools and daycare centres. In a study from Greece, 34 children with T1D using insulin pumps and CGM did not have a significant increase in time in range during lockdown but did have greater blood glucose variability when compared to the pre‐lockdown period.^49^ The children in this study were also noted to have dramatic changes in meal schedules during lockdown. Restrictions related to the COVID‐19 pandemic have resulted in decreased physical activity and dietary changes as well as altered diabetes management behaviours, all of which may increase risk of poor nutrition, excessive weight gain and increased stress related to diabetes management.^48^

Several studies have observed no difference in obesity rates between children with mild vs. severe COVID‐19 disease.^50^, ^51^, ^52^ While obesity in paediatric patients hospitalized for COVID‐19 is not more frequent than in the general paediatric population, COVID‐19 disease severity may be associated with obesity, as in adults. One report of 50 paediatric patients hospitalized with COVID‐19 identified obesity as a risk factor for mechanical ventilation.^53^ Furthermore, a recent multicentre study of COVID‐19 in 281 hospitalized patients under 22 years of age identified obesity (OR = 3.39, 95% CI: 1.26–9.10, *p* = .02) and hypoxia on admission as the only two underlying factors predictive of severe respiratory disease.^54^ In adults, worse outcomes related to obesity may be mediated by underlying cardiovascular and renal disease and hypertension.^55^, ^56^, ^57^

Children with metabolic bone disease or a skeletal dysplasia resulting in respiratory insufficiency due to altered chest wall structure may be at increased risk of COVID‐19 complications.^58^ Vitamin D modulates both innate and acquired immunity and may have direct antiviral effects via stimulation of antimicrobial peptides and promotion of autophagy; however, it is unclear if vitamin D deficiency increases the risk of COVID‐19 infection or complications. Some observational studies of adults have demonstrated lower serum 25‐OH‐vitamin D concentrations among patients infected with SARS‐CoV‐2 compared with controls, though data from the UK Biobank did not demonstrate an association of infection with serum 25‐OH‐vitamin D after adjustment for confounders.^59^, ^60^

## 4 MANAGEMENT OF ENDOCRINE DISORDERS IN CHILDREN IN THE COVID ERA

Management considerations for children and adolescents with underlying endocrine disorders, including those who develop COVID‐19 disease, is highlighted below. Special consideration should be given to prevent COVID‐19 infection in at risk populations. Approaches to management and treatment of paediatric endocrine disorders may have to be modified to decrease contact with healthcare team.

### 4.1 Hypopituitarism

Children with multiple pituitary hormone deficiencies may be at increased risk for COVID‐19 complications and mortality, particularly if central AI is not managed adequately. In the absence of published data or guidelines, we recommend following established practices for managing pituitary hormone deficiencies in children. Current guidance for adults with growth hormone deficiency recommends stopping growth hormone during hospitalization with COVID‐19; however, there is a lack of data regarding the effects of growth hormone treatment during COVID‐19 disease in children.^61^ Management of adrenal insufficiency is addressed below.

### 4.2 Central diabetes insipidus

Recommendations for management of central diabetes insipidus in patients with mild COVID‐19 disease do not vary from usual recommendations for management of diabetes insipidus in the home setting. However, patients of all ages with diabetes insipidus are at risk of disturbed sodium balance during hospitalization and must be monitored closely. Hypernatraemia can be caused by failure to administer free water to patients who are unable to care for themselves, and inability to rely on thirst mechanism in critically ill patients.^62^, ^63^ Patients are also vulnerable to hyponatraemia due to overtreatment of DI and excess ADH in the setting of COVID‐19 pneumonia. Treatment of DI with subcutaneous or oral desmopressin rather than intranasal desmopressin should be considered if there is concern for nasal congestion. In patients with severe COVID‐19 illness, desmopressin should be administered intravenously. Urine osmolality and volume should be monitored, and serum sodium should be measured at frequent intervals (every 2–4 h) to help maintain eunatraemia. Patients with COVID‐19 may have severe respiratory disease including pulmonary oedema, as hypernatraemia has not been implicated as a risk factor for mortality in COVID‐19, mild hypernatraemia may need to be tolerated under these circumstances to prevent pulmonary oedema.^31^

### 4.3 Primary adrenal insufficiency

Individuals who are steroid‐dependent or suspected of having adrenal suppression should, first and foremost, take caution to avoid SARS‐CoV‐2 infection. Patients should be managed according to existing guidelines regarding stress dosing during symptomatic COVID‐19 disease.^64^, ^65^

The RECOVERY trial, a randomized, open‐label trial of oral or intravenous dexamethasone (6 mg) daily vs. usual care, showed significant reduction in mortality in those receiving invasive mechanical ventilation and among those receiving oxygen without invasive mechanical ventilation, but not among those who were receiving no respiratory support at the time of randomization.^66^ In guidance released 2 September 2020 the World Health Organization made a strong recommendation for systemic corticosteroid therapy in patients with severe and critical COVID‐19, and a conditional recommendations not to use corticosteroid in patients with non‐severe COVID‐19.^67^

### 4.4 Diabetes mellitus

Viral illnesses can be more difficult to manage in individuals with diabetes due to increased insulin resistance and ketone production.^68^, ^69^ As higher A1C is positively correlated with frequency of DKA in children with T1D, a significant proportion of the paediatric T1D population may be at increased risk of developing DKA in the setting of COVID‐19 infection.^70^, ^71^ It is imperative that clinicians take the time to review sick day guidelines and be available for guidance during times of illness to reduce the risk of developing DKA.

During COVID‐19 illness, patients should be counselled to monitor blood glucose levels more frequently either via continuous glucose monitors or finger sticks.^72^, ^73^ Insulin doses may need to be titrated more often and additional correction boluses of fast‐acting insulin may be required to avoid severe hyperglycaemia and ketoacidosis.^74^ Patients should check ketones regardless of blood sugar levels and if ketones are present increase correction doses and fluid intake. Encouraging remote monitoring of blood glucose via CGM is a valuable tool that should be offered to all families.

For children with T2D, additional recommendations are like those addressed in the obesity section below. Of utmost importance is that all children maintain regular physical activity and strive for healthy eating habits during this pandemic.

### 4.5 Obesity

The shutting down of schools, camps and extracurricular sports and activities due to the pandemic has already had a profound impact on the health of children and adolescents due to social isolation, lack of activity and food insecurity in socioeconomically disadvantaged households.^75^, ^76^ In one of our centres, we have observed a significant increase in children under 19 years of age presenting with new‐onset diabetes, with most of the increase in new cases accounted for by an increase in type 2 diabetes (RM; unpublished data). It is more important than ever to reinforce healthy eating habits and provide age‐appropriate guidance on nutrition and physical activity. Racial/ethnic and socioeconomic disparities have heightened health inequities particularly related to weight management during the COVID‐19 pandemic. Healthcare providers should continue to provide education on healthy eating habits and regular exercise. We encourage access to telehealth interventions as adjuncts to paediatric weight management.

The risk of premature atherosclerotic cardiovascular disease (ASCVD) in youth who have had MIS‐C is not yet known; however, patients with a history of Kawasaki Disease with residual aneurysmal dilatation are considered high‐risk for ASCVD. In MIS‐C, coronary artery aneurisms have been found in 6%–24% of patients and Kawasaki disease features have been documented in up to 40%.^77^, ^78^ Recent data suggest that paediatric patients with MIS‐C who have been treated with IVIG or IL‐6 antagonists such as tocilizumab recover without sequelae.^79^ Given the unknown risk of complications, however, longer‐term cardiology follow‐up is warranted for children who have recovered from MIS‐C.

### 4.6 Metabolic bone disease

It remains to be determined whether vitamin D replacement will provide protection against COVID‐19 or complications. Randomized trials are in progress that will help determine whether vitamin D supplementation can prevent or decrease the severity of COVID‐19. Long periods of home quarantine designed to stop the spread of COVID‐19 may limit outdoor time, thus increasing the risk of vitamin D deficiency and associated complications including rickets, osteomalacia and symptomatic hypocalcaemia.^80^, ^81^ Clinicians should continue to follow current recommendations on vitamin D supplementation for patients at risk of deficiency. For patients with hypophosphatemic rickets, the FDA has recently approved home administration of burosumab injections during the COVID‐19 pandemic.

A joint statement by the American Society of Bone and Mineral Research, American Association of Clinical Endocrinology, Endocrine Society, European Calcified Tissue Society and National Osteoporosis Foundation offers guidance on the management of osteoporosis in adults during the COVID‐19 pandemic, some of which may need to be modified for paediatric patients. In children with low bone density, essential interventions to maintain bone health should be encouraged, including adequate intake of calcium and vitamin D and sex steroid replacement when indicated. Weight‐bearing activity is an essential component of optimizing bone health and should not be neglected; physical therapy services are now widely available via telemedicine and should be utilized to guide therapy in the home when needed. Advanced therapies should be continued when feasible, particularly in children who are at high risk of fragility fractures.

In the adult patient, due to the long‐acting nature of IV bisphosphonates, it is generally considered safe to delay treatment at least 6–9 months.^82^ However, in children, ongoing new bone growth and the occurrence of stress risers in the setting of intermittent bisphosphonate administration may increase fracture risk if treatment is substantially delayed.^83^ Consideration should be given to transitioning patients from pamidronate to zoledronic acid, which is infused over a shorter period of time and requires less frequent infusions. Clinicians can consider foregoing pre‐treatment laboratory testing for repeat infusions if the patient has no history of hypocalcaemia with previous infusions, is getting adequate calcium and vitamin D through the diet or supplementation, does not have any renal disease, and overall health status is stable. DXA scans and other imaging can be postponed in most patients when results of DXA scan will not change management.

### 4.7 Thyroid disease

Patients with primary hypothyroidism and hyperthyroidism disease should be managed per routine care. Anticipatory guidance should be given to patients taking anti‐thyroidal drugs (ATDs) such as methimazole, given the risk of agranulocytosis and secondary bacterial infection. Symptoms to monitor for while on ATDs—including fever, sore throat and cough—do overlap with symptoms of COVID‐19 thus prompt medical evaluation should be sought if symptoms arise. Additionally, like other infections, COVID‐19 may precipitate thyroid storm in patients with poorly controlled hyperthyroidism. Most patients with thyroid cancer are not at increased risk of infection given that surgical treatment with replacement levothyroxine is standard of care. For the rare patients on chemotherapy, there may be an increased risk of all infections due to immunosuppression. As per the American Thyroid Association and the American Association of Endocrine Surgeons, most thyroid cancer surgeries can be safely postponed without risk for worsening disease course given the typically slow tumour growth.^84^

## 5 CONCLUSION

From a mechanistic perspective, SARS‐CoV‐2 has the potential for disruption of most endocrine systems. While little is known about the interaction between COVID‐19 and endocrine disorders in the paediatric population, published data in adults have demonstrated pathology related to diabetes mellitus and obesity as well as pituitary, adrenal and thyroid disease. Current data highlight the following points related to COVID‐19 and endocrine disorders in children and adolescents:

- Treatment with anti‐thyroid medications and chronic corticosteroids is likely to increase risk of SARS‐CoV‐2 infection; patients should be counselled of this risk and take extra precautions to reduce risk of acquiring infection.
- MIS‐C appears to increase risk of hypocalcaemia in children, and those who are found to have developed coronary artery aneurisms may be at risk of later ASCVD.
- Children with T1D and higher A1C are more likely to be hospitalized with COVID‐19 than children with better diabetes control. The COVID‐19 pandemic has created a number of challenges in paediatric diabetes management related to school closures, disrupted schedules and stress related to diabetes management during periods of lockdown.
- Obesity does not appear to increase risk of acquiring SARS‐CoV‐2 infection in children, but obesity may be a risk factor for complications of COVID‐19 in this population.
- COVID‐19 in children with diabetes insipidus requires extra attention to fluid and sodium balance and may require a change in form of desmopressin delivery in those who are using the intranasal form.

We would also like to highlight the importance of recognizing indirect effects of the COVID‐19 pandemic on physical activity and eating habits that will worsen the problems of obesity, metabolic syndrome and associated complications due to shelter‐in‐home mandates and physical distancing restrictions. An acute increase in sedentary behaviour is known to cause decreased insulin sensitivity and elevations in blood glucose and over time increases the risk of incident T2D.^85^, ^86^, ^87^ We must continue to provide the support our patients require to minimize the health consequences of this pandemic on the next generation.

## CONFLICT OF INTEREST

A.G. has received consultancy fees from Medical Home Plus for Diabetes curriculum revision. A.L. has received research funding from Takeda Development Center Americas, Inc., Mannkind Corporation and the National Institute of Health. No financial interests or nonfinancial relationships have influenced his contribution to the manuscript. D.M. has pending consulting agreements with Chugai and Amolyt. All other authors have no competing interests to disclose.

## AUTHOR CONTRIBUTIONS

R.M. and S.K. jointly developed the outline and contributed to the writing and revision of the manuscript. M.K. and A.L. provided guidance on content and contributed to writing and critical review of the manuscript. A.A., E.G., A.G., B.K., D.M., S.M., D.M., N.P., V.R., D.R. and C.Y. each contributed to the writing, critical review and revision of this manuscript. All authors read and approved the final manuscript.

## Data Availability

Data sharing is not applicable to this article as no datasets were generated or analysed during the current study.
